# Treatments for irritable bowel syndrome: patients' attitudes and acceptability

**DOI:** 10.1186/1472-6882-8-65

**Published:** 2008-12-19

**Authors:** Lynsey R Harris, Lesley Roberts

**Affiliations:** 1 School of Medicine, C/O Lesley Roberts, Primary Care Clinical Sciences, University of Birmingham, Edgbaston, Birmingham, B15 2TT, UK; 2 Primary Care Clinical Sciences, University of Birmingham, Edgbaston, Birmingham, B15 2TT, UK

## Abstract

**Background:**

Irritable Bowel Syndrome, a highly prevalent chronic disorder, places significant burden on the health service and the individual. Symptomatic distress and reduced quality of life are compounded by few efficacious treatments available. As researchers continue to demonstrate the clinical efficacy of alternative therapies, it would be useful to gain a patient-perspective of treatment acceptability and identify patient's attitudes towards those modalities considered not acceptable.

**Methods:**

Six hundred and forty-five participants identified from an earlier IBS-prevalence study received a postal questionnaire to evaluate preferences and acceptability of nine forms of treatment. Proportions accepting each form of treatment were calculated and thematic analysis of qualitative data undertaken.

**Results:**

A total of 256 (39.7%) of 645 potential respondents completed the questionnaire (mean age 55.9 years, 73% female). Tablets were most acceptable (84%), followed by lifestyle changes (diet (82%), yoga (77%)). Acupuncture (59%) and suppositories (57%) were less acceptable.

When explaining lack of acceptability, patient views fell into four broad categories: dislike treatment modality, do not perceive benefit, general barriers and insufficient knowledge. Scepticism, lack of scientific rationale and fear of CAM were mentioned, although others expressed a dislike of conventional medical treatments. Past experiences, age and health concerns, and need for proof of efficacy were reported.

**Conclusion:**

Most patients were willing to accept various forms of treatment. However, the reservations expressed by this patient-population must be recognised with particular focus directed towards allaying fears and misconceptions, seeking further evidence base for certain therapies and incorporating physician support and advice.

## Background

Irritable Bowel Syndrome (IBS) is a chronic, relapsing, gastrointestinal disorder, affecting a substantial proportion of the population, with recent prevalence estimates between 10.5% and 30% [1-4]. IBS accounts for nearly 3% of general practitioner consultations^3 ^equating to approximately 1.8 million consultations in Britain per year[5]. Additionally it is known to have detrimental effects on quality of life [6]; effects which have been demonstrated as being similar to those of other common long-term medical disorders [7]. Symptom control is poor for a large proportion of patients. Thus it is understandable that failure of conventional treatment, the poorly understood pathology as well as the psychological components of IBS, have led to the development of complementary and alternative therapies targeted at symptom management.

Complementary and alternative medicine (CAM) includes all such practices and ideas outside the domain of conventional medicine and defined as preventing or treating illness, or promoting health and well-being [8]. It is known that chronic disease patients rank high among CAM users; 50.9% of IBS sufferers are reported to use CAM [9]. The popularity of CAM has grown significantly in all modern societies over the past two decades [10], demonstrated by an increase in general practices offering access to CAM, 49% in 2001 compared to 39% in 1995 [11]. The Department of Health (UK) has also recognised its growing significance, particularly in relation to primary care services. Their 2003 policy document includes recommendations for developing a framework facilitating patient access to CAM [12].

It is likely that patients have clearly defined preferences for treatments, both in terms of modality and general theoretical perspective and these preferences may differ between different subgroups of patients. In western countries, CAM use is reported to be more frequent in women, well-educated individuals, and amongst middle-aged rather than elderly people [13]. One could assume that CAM is seen as a 'last resort', as many studies have shown that CAM use is associated with a long illness duration, poor functional status and co- morbidity [14,15]. However, a recent study which controlled for other confounders did not support this [16], suggesting that for some individuals CAM is a preferred primary management strategy. It is possible that this will apply more so for conditions perceived as 'non-threatening' but causing significant disruption to everyday life.

It is not clear to what extent treatment acceptability is affected by its recommendation by a doctor or health professional. It has been reported that a large proportion of patients (62%) prefer their doctors to discuss non-evidence based treatment options with them, primarily to offer advice but also in case of interactions with other medicines [17]. Although this is not always the case, some patient groups may prefer the medical team to make treatment decisions on their behalf [18]. It is possible that treatments advised by the doctor may be perceived as more credible, emphasising the importance of health professionals' knowledge and understanding of different treatment options.

Hypnotherapy and relaxation strategies have been shown to be effective in clinical trials [19,20] and there is evidence to support efficacy for some forms of herbal therapy and certain probiotics in IBS [21,22]. However, many patients still seem adverse to alternative management strategies [9]. Obviously outcome is not exclusively important to patients; acceptability may also depend on the nature and form of delivery of therapy. Promising therapies may not be worth pursuing if they are unacceptable to the majority of patients. Reasons for this have been under-researched in patients with chronic non-threatening disease. To date, no information is available on the attitudes and acceptability of different therapies and modes of delivery in relation to IBS patients. The primary aim of this study was to explore the above, and the secondary aim was to determine whether a patient's willingness to accept a treatment for their IBS is dependent on the recommendation of a doctor or related to patient characteristics such as age, gender, education level attained or employment. Findings from this study will not only offer the health professional insight but also complement existing knowledge on the efficacy and costs of different treatments to guide therapeutic development.

## Methods

### Ethical approval

Ethical Approval was granted through the Wolverhampton Local Research Ethics Committee (Ref 05/QZ701/86).

### Setting/sample

In 2000/2001 a large study was conducted in Birmingham, to establish the prevalence of IBS in the community [1]. A postal questionnaire was sent to 8646 patients aged 18 and over, who were randomly selected from the registers of eight general practices in North and West Birmingham. Of those who responded, 1151 (n = 4807) patients were identified as having a Rome II diagnosis (n = 398) [23] or two or more symptoms suggestive of IBS (according to Rome II) (n = 753). Of these, 892 individuals responded to an invitation to provide symptom data.

This cohort of patients (n = 892) was followed up in 2006 to explore the epidemiological trends and establish the natural history of IBS. Patients were excluded (n = 247) due to terminal illness or other circumstance in which the GP felt contact was inappropriate (n = 15), including a recent more significant bowel diagnosis, where they had transferred GP practice, (so current status could not be confirmed) (n = 193), or where they were known to be deceased (n = 39). The 645 remaining patients were re-contacted and invited to complete a further postal questionnaire [see Additional file 1], which collected data about acceptability of a range of treatment options including both traditional and conventional forms of delivery and complementary and alternative strategies.

The sample size was limited to responders of previous questionnaires; therefore no formal sample size calculation was performed.

### Intervention

A questionnaire and free-post return envelope were mailed to patients. Subjects not wishing to take part in the study were notified to return a blank copy to avoid further contact while those failing to respond within 21 days were sent another questionnaire as a reminder. Patients were invited to complete the questionnaire as fully as possible although were not obligated to do so. Questionnaires did not contain identifiable data but were coded (and therefore not anonymous) for administrative purposes. All participants were informed of the purpose of the study and return of a completed questionnaire was deemed to imply consent.

### Outcome measures

Each patient received an identical two-part questionnaire. The first part elicited information about their IBS symptoms and demographic details including age, gender, ethnicity, and education level. The second part addressed their attitudes and acceptability of various treatment options. Nine types of treatment options which reflected diverse delivery modalities and a range of underlying belief systems were presented; taking a tablet (drug therapy), applying a cream to stomach, suppositories, exercises (similar to yoga), diet change, acupuncture, hypnotherapy, homeopathy, and application of a heat pad. Patients were asked whether they would consider each of these treatments under three different scenarios a) if their doctor recommended the treatment as part of their usual medical care, b) if the treatment was offered as part of a research study, or c) if they had to arrange and pay for the treatment themselves. If they accepted the treatment under any one of these scenarios they were categorised as accepting that modality. If they did not find the treatment acceptable, they were encouraged to detail in a free text space their reasons.

Questionnaires were reviewed for completeness and responses entered into a Microsoft Access database. For the purposes of data entry validation, 10% of the data was dual entered.

### Analysis

Simple description of demographic features of the respondents and the proportion of patients willing to use each type and form of treatment was undertaken. Chi-squared tests were performed to determine whether acceptability was related to personal characteristics; age, gender, educational achievement and employment status. As this involved multiple testing, statistical significance was considered if p < 0.01. Age was collapsed into two groups based on group median age (≤55 years and >55 years). Education was categorised as working 20+ hours per week, working <20 hours per week, and retired/other. Educational attainment was classified as leaving school at or before 16 years of age or schooling beyond age 16. Ethnicity was not included in the analysis as there were too few numbers for ethnicities other than Caucasian to allow statistical testing. Statistical analyses were performed using SPSS for Windows version 13.0.

Qualitative analysis of written responses in the questionnaires was undertaken with identification of themes emerging from data relating to reasons for therapeutic refusal being the primary objective of analysis. A manual indexing system was applied to all data and eventually major organising themes were identified to reflect patients' attitudes to the acceptability of different treatments. Qualitative data was initially coded by one researcher (LH) with a second researcher (LR) subsequently reviewing the emerging themes with reference to the full data.

## Results

### Demographic data

Of the 645 patient cohort, 377 responded (58.4%), however 121 questionnaires were returned with this section of the questionnaire unanswered; therefore an overall 39.7% (n = 256) response rate was achieved. Of the responders, 94% were Caucasian, 73% female, and average age was 56 years. Demographics of those who returned the questionnaire were similar to those who did not (Table 1).

**Table 1:** Demographic information

	Respondents (n = 256)	Non-respondents (n = 389)	P value
Mean age +/- s.d.	55.9 +/- 14.8 years	54.6 +/- 17.4 years	0.30
			
**Sex**			
Female	188 (73.4%)	274 (70.0%)	0.46
Male	68 (26.6%)	115 (30.0%)	
**Race**			
Caucasian	241 (94.1%)	354 (91.0%)	0.19
other	15 (5.9%)	35 (9.0%)	
**Highest education level achieved**		Not known – may have changed since baseline data collected	N/A
No school or Primary school	10 (3.9%)		
Secondary school	168 (65.6%)		
University/higher education	77 (30.1%)		
			
**Employment status**			
Part time/full time work	135 (52.8%)	Not known – may have changed since baseline data collected	N/A
Full time home maker	23 (9.0%)		
Retired	62 (24.2%)		
Not working: unemployed or due to poor health	33 (12.9%)		
Other	3 (1.2%)		

### Treatment preferences and acceptability

Table 2 displays the number of patients accepting each treatment modality. The greatest number of patients accepted medication as a tablet form (84%) followed by a diet change (82%) and yoga (77%). Seventy six individuals were accepting of all modalities and only ten participants indicated none of the proposed treatment forms was acceptable to them. Acupuncture (59%) and suppositories (57%) were less accepted. Acceptability of a treatment was greatest in all cases when recommended by a clinician.

**Table 2:** Number of patients accepting each treatment modality (n = 256)

**Type of treatment**	**Accept the treatment under at least 1 of the circumstances**	**Proportion accepting treatment (95% CI)**
Tablets	215	83.9% (79.4,88.4)
Diet change	209	81.6% (76.9,86.4)
Yoga	196	76.6% (71.4,81.8)
Stomach cream	173	67.6% (61.9,73.3)
Homeopathy	166	64.8% (59.0, 70.7)
Heat pad	163	63.7% (57.8,69.6)
Hypnotherapy	163	63.7% (57.8,69.6)
Acupuncture	151	59.0% (53.0,65.0)
Suppository	147	57.4% (51.3, 63.5)

Age was related to accepting yoga (p = 0.01), diet change (p = 0.02) and hypnotherapy (p = 0.02) but not the other treatment modalities, with younger respondents (55 years and under) being more likely to accept such treatment forms than older respondents.

Gender was not significantly related to accepting any form of treatment, nor was education level or employment status.

When categorising treatments into conventional treatments (tablets, suppositories, creams and heat pad), lifestyle (exercises like yoga, diet change) and CAM (acupuncture, hypnotherapy, homeopathy), age was again significantly related to accepting treatments, with younger patients (age ≤55 years) being more accepting of lifestyle changes (p = 0.006). Age was not significantly associated at the p < 0.01 level with acceptance of conventional forms of treatment (p = 0.049) or CAM (p = 0.11).

### Qualitative analysis

#### Reasons for finding a treatment unacceptable

**Figure 1:**
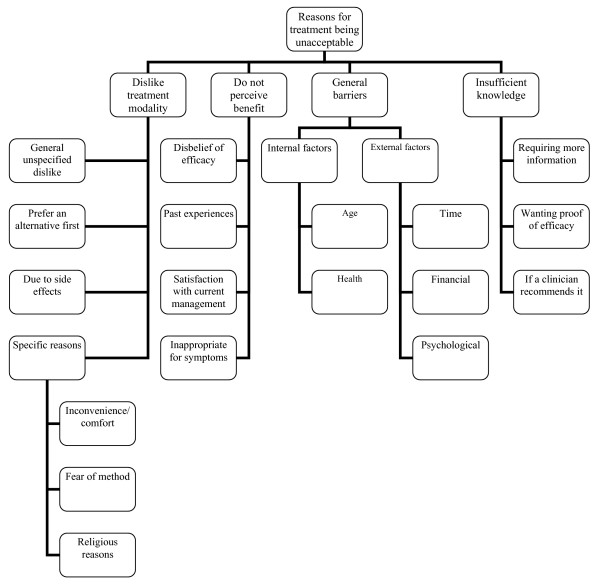
**Reasons for treatment *unacceptability***.

The themes and sub themes that emerged from the data are displayed in figure 1. The four main themes are broadly defined as:

1. Dislike treatment modality

2. Do not perceive benefit

3. General barriers (internal factors for example ill health and external factors such as time and accessibility)

4. Insufficient knowledge

**Figure 2:**
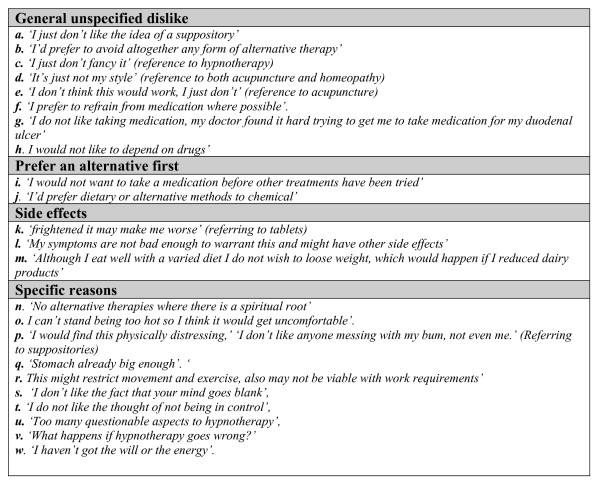
**Quotes illustrating theme 1: dislike treatment modality**.

**Figure 3:**
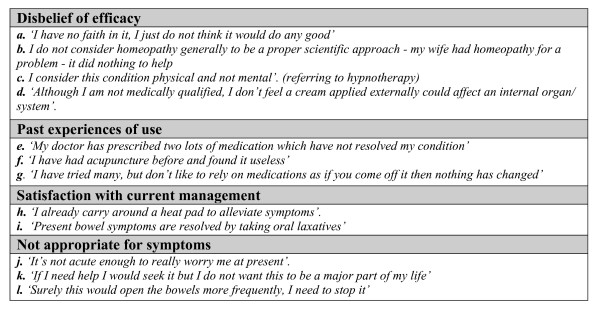
**Quotes illustrating theme 2: do not perceive benefit**.

**Figure 4:**
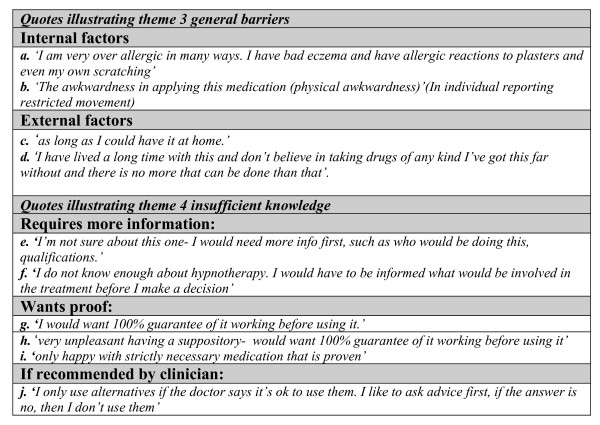
**Quotes illustrating themes 3 & 4: general barriers to acceptance and insufficient knowledge**.

Themes are supported by patient responses. Due to the questionnaire nature of this study these were mainly short responses, and have therefore been presented in figures 2, 3 and 4 to prevent disruption of the main body of text. Supporting quotes are referenced in the text as figure number followed by quote index.

### Dislike treatment modality

#### General dislike

Regardless of which treatment was chosen, there were some patients who expressed a general non-specified dislike for it, ^2a,2b ^although this seemed more evident in reference to hypnotherapy, homeopathy and suppositories.^2c,2d,2e ^Many individuals held fixed preconceptions against such non-mainstream therapies which they did not expand upon.

This theme was also strongly portrayed in relation to tablet medication; some subjects were wary of taking tablets and preferred to avoid them. ^2f,2g ^Tablets were also seen as being associated with reliance and dependence. ^2h^

#### Prefer an alternative first

Emerging initially in the views of those disliking tablets there was a group of individuals who expressed the view that the unacceptability of some treatments was due to their preference for 'non-medical alternatives' as initial management. This theme was evident amongst individuals rejecting any of the conventional forms of therapy (tablets, suppositories and creams). This may be because some perceive IBS as having dietary or psychosocial elements rather than a 'medical' cause. ^2i,2j^

#### Dislike due to side effects

The risk of possible side effects was enough to deter some patients.^2k,2l ^In the case of tablets, this could be because they are ingested and therefore perceived as having an internal action with potential positive or negative effects.

Side effects were also mentioned as reasons to avoid creams and suppositories although to a lesser extent than for tablets. It therefore appeared that treatments perceived as 'conventional' were related to a perception of greater risks of side effects.

A diet change was positively regarded as a lifestyle management option, although this also carried with it some fear of side effects; the risk of losing weight being one reason why individuals did not find it acceptable. ^2m^

#### Reasons which relate to specific treatment methods

Some individuals regarded homeopathy, yoga, acupuncture and hypnotherapy as having a spiritual association and for religious reasons found these types of treatments unacceptable. ^2n^

Personal comfort is particularly important for individuals when evaluating and considering treatment methods. ^2o,2p ^An underlying theme common amongst yoga, hypnotherapy and suppositories was embarrassment and the existence of personal tolerance thresholds. Suppositories and stomach creams were also considered to be invasive and messy. Although heat pads were generally found to be acceptable, some individuals described problems of convenience, practicality and appearance. ^2q,2r^

Patients described how they were frightened by some of the treatments. These views centred on hypnotherapy and acupuncture. ^2s,2t,2u,2v ^The main hesitance associated with acupuncture was an overwhelming fear of needles and pain.

Some treatment modalities, such as yoga, were perceived to require greater personal commitment, effort, and may be inconvenient to daily life. ^2w^

### Do not perceive benefit

#### Disbelief of efficacy

A particularly strong theme evident amongst acupuncture, homeopathy, and hypnotherapy was a general scepticism and disbelief in their effectiveness.^3a,3b,3c ^Heat pads and stomach cream were also regarded with some scepticism due to their external application which some felt was at odds with management of an 'internal' problem.^3d ^Perceptions of efficacy were stronger for conventional style medications, which could be related to greater knowledge and experience of mainstream therapies.

#### Past experiences of use

Past experiences and poor outcomes shaped many patients attitudes. ^3e,3f,3g^

#### Satisfaction with current management

Those patients satisfied with current treatment did not feel the need to change their practice. ^3h,3i^

#### Not appropriate for symptoms

A treatment was considered inappropriate for one of two reasons. The first was if the individual's symptoms were not considered severe enough. ^3j,3k ^Some therapies may be perceived as having more associated risks, and would only be considered if symptoms were very severe. The second reason suggested was related to known associations between treatment types and symptoms. For example, suppositories were perceived as useful in the management of constipation but not with diarrhoea. ^3l^

### General barriers to acceptance

#### Internal factors

Some patients viewed other health problems as a barrier to using certain therapies, for example co-existing skin disorders such as eczema, psoriasis, and skin allergies were mentioned as contra-indicating use of creams and heat pads. ^4a^

Many health restraints were mentioned in relation to yoga: pregnancy, poor flexibility, being physically unfit and a number of back and joint problems. Spine problems, past operations and paraesthesia discouraged some individuals from considering acupuncture. Health restraints for inserting a suppository included anal fissures, piles, arthritis, incontinence and pain in back and legs. ^4b^

Age was also used to explain aversion to some therapies. Being too old was stated as a barrier to yoga therapy. Age was also indicated to impact on flexibility essential for applying creams and suppositories and thus the age of the target population for therapies should certainly be considered.

#### External factors

Other barriers to treatments included time, financial costs and access. Tablets and creams were generally perceived as being reasonably expensive whilst acupuncture, hypnotherapy and yoga as both expensive and time consuming. In some instances accessibility was the only deterring factor. ^4c^

#### Psychological

Resignation seemed to play a part for some individuals. ^4d ^Some individuals were prepared to accept their symptoms, without seeking relief or cure.

### Insufficient knowledge

#### Requiring more information

Many subjects felt they required more information before they could make a decision regarding treatments. This applied primarily to ingested therapies (tablets, homeopathic tablets) but did also apply to most treatments with an unknown element such as hypnotherapy. ^4e,4f^

#### Wanting proof of efficacy

Requiring proof of efficacy was an important and repeated theme.^4g,4h,4i^Interestingly this theme emerged more strongly amongst the conventional treatments such as tablets and suppositories. This may be due to the greater perceived risks of these more intrusive forms of treatment.

#### If recommended by clinician

Most subjects were more accepting of CAM treatments if recommended by a clinician.^4j ^This is likely to be due to a combination of factors related to above themes including an assumption of efficacy if advised by a clinician, provision of information about the therapy including side effects or interactions with concomitant medication, and individual preference for clinician directed disease management.

## Discussion

Quantitative analyses indicated that the treatments most strongly endorsed were those most closely aligned with conventional medicine, notably tablets. Lifestyle changes are more favourable than CAM suggesting that preference for 'a quick fix' is not a primary driver in the choice of therapy. These findings are supported by other literature which has shown that patients perceive diet and prescription medicines to have the greatest benefit for their IBS [24]. Perhaps lifestyle changes are believed to have fewer risks and side effects which deterred patients from other methods of treatment, and with this style of treatment, patients have greatest control. As patient empowerment is increasingly more important in modern medical practice, this could be an area to target within IBS management.

All treatments were accepted by more than half the study population, supporting the current level of unmet need and desire to explore therapeutic options in this patient group. Research in another area of chronic disease management (chronic pain) reported similar findings; a high proportion of patients willing to try exercise and interested in learning relaxation techniques to alleviate symptoms [25].

In general, these results do not support prior studies suggesting well-educated, middle aged women are more accepting of CAM [13]. This may be because this study presented the options as potentially available and therefore obtained a better view of acceptability if barriers of access and cost are removed. However, age was significantly associated with willingness to accept some of the treatments with younger patients (<56 years) being more inclined to accept yoga, hypnotherapy and dietary change.

Gender was not associated with willingness to accept any therapies, in contrast to other studies [13], although this finding may be attributable to sample size and dominance of female respondents (73%).

The qualitative data suggest that the three forms of treatment modalities present unique barriers to acceptance. Conventional modalities, (tablets, suppositories, creams) were rejected as their association with mainstream treatments gives a greater perception of risk and possibility of side effects. Those who perceived their symptoms as less severe were less accepting of treatments they believed to be 'risky' in line with other literature suggesting less severe symptoms predicts treatment disuse [26].

Conversely CAM modalities may be preferred as they are 'less risky' but were viewed with scepticism, drawing a parallel with other literature [26]. Patients wanted more information and proof of efficacy. Specific modalities frightened some individuals; fear of needles and hypnotism were quoted. Spiritual associations deterred some individuals who felt this conflicted with their religious beliefs. Lifestyle changes were generally well accepted; although require more effort for the individual.

For some individuals time and financial costs were central to acceptability, as previous research suggests [25]. For treatments that are more provider-based as opposed to self-care based, perceived lack of accessibility to providers also explained unacceptability. Due to the older average age of this population, age-related health concerns also featured significantly amongst potential barriers. For skin-contactable treatments such as creams and heat pads, skin disorders and allergies were potential barriers. Psychological barriers also emerged from this study. Patients who were resigned to their symptoms were less willing to try treatments. Hence psychological aspects and patient cooperation must be considered.

Results from this study showed that patients were more inclined to accept any of the treatments if recommended by a physician, reflecting the value assigned to clinician support. This suggests members of the clinical team may be ideally positioned to explore patient reservations and provide education and evidence. Lack of physician support has been statistically significantly associated with CAM disuse in other studies [26].

### Study limitations

As this data was collected as part of a larger epidemiological study and utilised to form a secondary exploratory study, it is limited by the data available and the population demographics of those included.

There were a number of limitations to the study. Firstly, respondents were mainly Caucasian, and thus data may not be applicable to other ethnic populations. Also the limited range of ethnicities and female predominance limited the statistical ability to compare these. Over simplification of other categories, eg: age, may mean that differences between groups have not been identified, but were undertaken to enable comparative analyses. Secondly, a 40% response rate is quite low. This may be explained by the fluctuating nature of IBS; during some time periods individuals may be symptom free which may suggest a lower level of commitment to survey research of this type. A large loss to follow-up reduced the size of the cohort. However, there was a large initial sample size and similar demographics between responders and non-responders. It has been acknowledged that a larger qualitative study with a broader sample of patients from a diverse range of settings could have enhanced the quality of the data. The overwhelming female representation in this cohort could limit generalisability of these findings to male patient groups. Given the greater prevalence of IBS in females the findings of this study are likely to be representative of an IBS population.

As with all qualitative analyses, the introduction of bias by the researcher remains a possibility. Such bias is believed to be further reduced by the different experiential backgrounds on which researchers drew (one clinical and one non-clinical, and only one with personal experience of IBS). It is also acknowledged that qualitative analysis from written responses may introduce bias as some individuals may find it hard to articulate their views, responses may have been misinterpreted, or the defined space for comments may have limited respondents.

## Conclusion

As acknowledged, IBS represents significant demands on the health service and is a source of considerable frustration amongst patients and health care professionals. Many reasons for which, relate to the absence of universally accepted and effective treatments. As researchers continue to demonstrate the clinical efficacy of CAM therapies, these findings deserve attention because they highlight potential reasons why patients may be biased against such therapies. These findings provide a basis for further research on possible types of treatments with a long term objective of developing acceptable and effective interventions for IBS sufferers.

The study demonstrated that most patients were interested in trying therapeutic options that lie outside the conventional medical spectrum, but a substantial number of barriers to these were also outlined. From these it seems that addressing gaps in patient's knowledge, overcoming specific fears and applying evidence based knowledge whilst working in conjunction with physicians could improve management for IBS and subsequently patient quality of life. Further work in this area, using interview methods is recommended to more fully explore patient perceptions and decision making processes.

## Competing interests

The authors declare that they have no competing interests.

## Authors' contributions

LH carried out the acquisition of data, analysis and interpretation of data, drafted and revised the manuscript and approved the final version to be published.

LR conceived and designed the study, carried out the acquisition of data, revised the manuscript and approved the final version to be published.

## Pre-publication history

The pre-publication history for this paper can be accessed here:



## Supplementary Material

Additional file 1 **IBS Cohort Follow-up Questionnaire.** The file represents the questionnaire which was issued to participants for this survey. The data reported in this paper refers to that collected from section 5 of this questionnaire. Click here for file
