# Supplementary material for: Treatments for irritable bowel syndrome: patients' attitudes and acceptability
Source: BMC Complement Altern Med. 2008 Dec 19;8:65. doi: 10.1186/1472-6882-8-65 (PMC2633319; doi:10.1186/1472-6882-8-65)
Supplement: Additional file 1 — IBS Cohort Follow-up Questionnaire. The file represents the questionnaire which was issued to participants for this survey. The data reported in this paper refers to that collected from section 5 of this questionnaire. [file 1472-6882-8-65-S1.doc]

# HEALTH QUESTIONNAIRE

A few years ago you took part in a health survey, which identified that at you were experiencing abdominal (stomach) or bowel symptoms or had a diagnosis of Irritable Bowel Syndrome. Thank you again for your help in this survey.

We would now like to follow-up the people who took part in this survey to identify whether their abdominal pain has improved or worsened over time. Knowing this will help us to identify the things that are related to long-term illness and will help in the development of treatments for patients with Irritable Bowel Syndrome or other abdominal pain.

Recent research suggests that some of the things that are important to patients with abdominal and bowel symptoms are not usually measured in research studies. For example, doctors often ask about pain, but you may think that increasing your confidence to go out despite symptoms is more important to you. We would therefore be grateful if you could also help by answering some questions about what things are important to you and the types of treatments you would consider. Again this will help us design research that fits the needs of patients.

**Even if you no longer have any abdominal or bowel symptoms, your response is very important to us.**

It takes most people about 15 minutes to complete this questionnaire. Many of the questions just require you to tick a box as a response. Other questions ask you to write how you feel. These questions may seem a little harder to complete than tick boxes but they give the opportunity for you to explain the things that are important to you and we would like to encourage you to think about these questions and complete them if you can. It is important that we obtain as much information as possible, so we ask that you try to answer every question**.** However, if you do not wish to complete some of the questions please leave them blank. We would rather you answered some of the questions than none of them.

If you decide not to participate in this research, please send back the questionnaire in the FREEPOST envelope provided rather than throwing it in the bin. If you do this we will respect your privacy and we will not contact you again.

| Thank you for your co-operation.  Please return this questionnaire in the **FREEPOST** envelope provided.  No stamp is required. |
| --- |

|  | Dr. Lesley Roberts  Department of Primary Care & General Practice  The Medical School  The University of Birmingham  Edgbaston  Birmingham B15 2TT  Telephone 0121-414-3356 |
| --- | --- |

**Please give us details of your age and sex below:**

| What is your date of birth? |  |  |
| --- | --- | --- |

| What is your sex? | Male | **□** | or | Female | **□** |
| --- | --- | --- | --- | --- | --- |

**SECTION 1.**

**The questions in this section ask about your current abdominal and bowel symptoms and any new diagnoses you may have. Some of these questions are the same as you answered last time we wrote you to. You need to tell us about your symptoms now.**

1. **Has a doctor ever told you that you have any of the conditions below?**

**Please tick either ‘Yes’ or ‘No’ for each condition. If the answer is ‘Yes’ please let us know when this was diagnosed. If you cannot remember please give us your best guess (e.g. March 2004, Summer 1998, 1970, Early 1950’s)**

|  | Has a doctor ever told **you** that you have or had this condition? | If ‘Yes’ please tell us when this condition was diagnosed |
| --- | --- | --- |
| Depression / Anxiety | Yes **□** No **□** |  |
| Liver problems | Yes **□** No **□** |  |
| Kidney stones | Yes **□** No **□** |  |
| Stomach ulcer | Yes **□** No **□** |  |
| Irritable bowel syndrome | Yes **□** No **□** |  |
| Ulcerative colitis | Yes **□** No **□** |  |
| Crohn’s disease | Yes **□** No **□** |  |
| Inflammatory bowel disease | Yes **□** No **□** |  |
| Indigestion / Dyspepsia | Yes **□** No **□** |  |
| Duodenal / Peptic ulcer | Yes **□** No **□** |  |
| Any other disease of the stomach or gastrointestinal tract (your food tract)  *If yes please specify* | Yes **□** No **□** *__________________________________________* |  |
| Cancer *If yes, please specify:* | Yes **□** No **□** *__________________________________________* |  |

**2. Please tick either ‘Yes’ or ‘No’ to the questions below which ask about your abdominal pain and bowels. Some of these questions ask about passing a stool. By this we mean going to the toilet for a reason other than to urinate (pass water).**

| **2.1** | |  | YES | NO |
| --- | --- | --- | --- | --- |
|  | Do you ever suffer from troublesome abdominal (belly or tummy) pain? | | **□** | **□** |
|  | During the last year have you had 12 weeks or more of abdominal pain or discomfort? This doesn’t need to be consecutive weeks (but overall 12 weeks or more) | | **□** | **□** |

If you have answered ‘No’ to BOTH of the questions above, please go to section 2.3 below.

If you answered ‘Yes’ to EITHER of the questions above, please answer section 2.2 and 2.3.

| **2.2**  **Think about the times when you have abdominal pain or discomfort:** | | YES | NO |
| --- | --- | --- | --- |
|  | Is your abdominal pain relieved or helped by opening your bowels to pass a stool? | **□** | **□** |
|  | When you get your pain do you notice a change in stool frequency (going more or less often than usual)? | **□** | **□** |
|  | When you get your pain do you notice a change in the appearance of your stool (either more runny, lumpy, harder etc.)? | **□** | **□** |

| **2.3 Do you experience any of the following:** | | YES | NO |
| --- | --- | --- | --- |
|  | Pass stools more than 3 times a day | **□** | **□** |
|  | Pass stools less than 3 times a week | **□** | **□** |
|  | Abnormal stools (lumpy, hard, watery, etc) | **□** | **□** |
|  | Need to strain to pass a stool | **□** | **□** |
|  | Feeling of urgency (feeling that you must immediately rush to the toilet to pass a stool) | **□** | **□** |
|  | Feeling of incomplete evacuation (feeling that after finishing a bowel movement there was still a stool which needed to be passed) | **□** | **□** |
|  | Passage of slime or mucus | **□** | **□** |
|  | Bloating or swelling of the abdomen (tummy) | **□** | **□** |

| **2.4** | **YES** | **NO** |
| --- | --- | --- |
| Do you take any medication for your abdominal or bowel symptoms? | **□** | **□** |
| **If you answered YES to the above question**,  How many days per week do you require this medication? |  | |

**SECTION 2.**

**The following questions ask you about abdominal and bowel symptoms. When we use the word abdomen we mean belly/tummy. All of these questions refer to the last 4 weeks. Even if you no longer experience abdominal pain please complete this section.**

**Please tick one box for each question.**

|  |  | **All of the time** | **Most of the time** | **A good bit of the time** | **Some of the time** | **A little of the time** | **None of the time** |  |
| --- | --- | --- | --- | --- | --- | --- | --- | --- |
| **1.** | During the last 4 weeks, how often have you had discomfort or pain in your abdomen? | **□** | **□** | **□** | **□** | **□** | **□** |  |
| **2.** | How often have you been troubled with loose, mushy or watery bowel motions during the last 4 weeks? | **□** | **□** | **□** | **□** | **□** | **□** |  |
| **3.** | How often during the last 4 weeks have you been troubled with diarrhoea? | **□** | **□** | **□** | **□** | **□** | **□** |  |
| **4.** | During the last 4 weeks how often have you been troubled by hard bowel motions? | **□** | **□** | **□** | **□** | **□** | **□** |  |
| **5.** | During the last 4 weeks how often have you felt the need to strain to pass a motion (stool)? | **□** | **□** | **□** | **□** | **□** | **□** |  |
| **6.** | During the last 4 weeks how often have you been troubled by constipation? | **□** | **□** | **□** | **□** | **□** | **□** |  |
| **7.** | During the last 4 weeks how often did you experience pain or discomfort in your abdomen after eating? | **□** | **□** | **□** | **□** | **□** | **□** |  |

|  |  | **All of the time** | **Most of the time** | **A good bit of the time** | **Some of the time** | **A little of the time** | **None of the time** |  |
| --- | --- | --- | --- | --- | --- | --- | --- | --- |
| **8.** | How often has your abdominal pain prevented you from sleeping, or woken you during the last 4 weeks? | **□** | **□** | **□** | **□** | **□** | **□** |  |
| **9.** | During the last 4 weeks how often have you leaked or soiled yourself? | **□** | **□** | **□** | **□** | **□** | **□** |  |
| **10.** | How often during the last 4 weeks have you suffered from a feeling of urgency (feeling that you must immediately rush to the toilet to pass a stool)? | **□** | **□** | **□** | **□** | **□** | **□** |  |
| **11.** | How often have you passed mucus or slime in your stools over the last 4 weeks? | **□** | **□** | **□** | **□** | **□** | **□** |  |

**SECTION 3:**

**This section is designed to help us understand how your abdominal pain or bowel symptoms affect your everyday life. Please try to answer all of the questions and complete this section even if you no longer experience abdominal symptoms. Tick ONE box for each statement.**

|  |  | **Not at all** | **Slightly** | **Moderately** | **Quite a bit** | **Extremely/ a great deal** |  |
| --- | --- | --- | --- | --- | --- | --- | --- |
| **1.** | I feel helpless because of my abdominal/bowel problems. | **□** | **□** | **□** | **□** | **□** |  |
| **2.** | I am embarrassed by the smell caused by my abdominal/bowel problems. | **□** | **□** | **□** | **□** | **□** |  |
| **3.** | I am bothered by how much time I spend on the toilet. | **□** | **□** | **□** | **□** | **□** |  |
| **4.** | I feel vulnerable to other illnesses because of my abdominal/bowel problems. | **□** | **□** | **□** | **□** | **□** |  |
| **5.** | I feel fat because of my abdominal/bowel problems | **□** | **□** | **□** | **□** | **□** |  |
| **6.** | I feel like I’m losing control of my life because of my abdominal/bowel problems. | **□** | **□** | **□** | **□** | **□** |  |
| **7.** | I feel my life is less enjoyable because of my abdominal/bowel problems. | **□** | **□** | **□** | **□** | **□** |  |
| **8.** | I feel uncomfortable when I talk about my abdominal/bowel problems. | **□** | **□** | **□** | **□** | **□** |  |
| **9.** | I feel depressed about my abdominal/bowel problems. | **□** | **□** | **□** | **□** | **□** |  |
| **10.** | I feel isolated from others because of my abdominal/bowel problems. | **□** | **□** | **□** | **□** | **□** |  |
| **11.** | I have to watch the amount of food I eat because of my abdominal/bowel problems. | **□** | **□** | **□** | **□** | **□** |  |

|  |  | **Not at all** | **Slightly** | **Moderately** | **Quite a bit** | **Extremely/ a great deal** |  |
| --- | --- | --- | --- | --- | --- | --- | --- |
| **12.** | Because of my abdominal/ bowel problems, sexual activity is difficult for me. | **□** | **□** | **□** | **□** | **□** |  |
| **13.** | I feel angry that I have abdominal/bowel problems. | **□** | **□** | **□** | **□** | **□** |  |
| **14.** | I feel that I irritate others because of my abdominal/ bowel problems. | **□** | **□** | **□** | **□** | **□** |  |
| **15.** | I worry that my abdominal/ bowel problems will get worse. | **□** | **□** | **□** | **□** | **□** |  |
| **16.** | I feel irritable because of my abdominal/bowel problems. | **□** | **□** | **□** | **□** | **□** |  |
| **17.** | I worry that people think I exaggerate my abdominal/ bowel problems. | **□** | **□** | **□** | **□** | **□** |  |
| **18.** | I feel that I get less done because of my abdominal/ bowel problems. | **□** | **□** | **□** | **□** | **□** |  |
| **19.** | I have to avoid stressful situations because of my abdominal/bowel problems. | **□** | **□** | **□** | **□** | **□** |  |
| **20.** | My abdominal/bowel problems reduce my sexual desire. | **□** | **□** | **□** | **□** | **□** |  |
| **21.** | My abdominal/bowel problems limit what I can wear. | **□** | **□** | **□** | **□** | **□** |  |
| **22.** | I have to avoid strenuous activity because of my abdominal/bowel problems. | **□** | **□** | **□** | **□** | **□** |  |
| **23.** | I have to watch the kind of food I eat because of my abdominal/ bowel problems. | **□** | **□** | **□** | **□** | **□** |  |

|  |  | **Not at all** | **Slightly** | **Moderately** | **Quite a bit** | **Extremely/ a great deal** |  |
| --- | --- | --- | --- | --- | --- | --- | --- |
| **24.** | Because of my abdominal/ bowel problems, I have difficulty being around people I do not know well. | **□** | **□** | **□** | **□** | **□** |  |
| **25.** | I feel sluggish because of my abdominal/bowel problems. | **□** | **□** | **□** | **□** | **□** |  |
| **26.** | I feel unclean because of my abdominal/bowel problems. | **□** | **□** | **□** | **□** | **□** |  |
| **27.** | Long trips are difficult for me because of my abdominal/ bowel problems. | **□** | **□** | **□** | **□** | **□** |  |
| **28.** | I feel frustrated that I cannot eat when I want to because of my abdominal/bowel problems. | **□** | **□** | **□** | **□** | **□** |  |
| **29.** | It is important to be near a toilet because of my abdominal/bowel problems. | **□** | **□** | **□** | **□** | **□** |  |
| **30.** | My life revolves around my abdominal/bowel problems. | **□** | **□** | **□** | **□** | **□** |  |
| **31.** | I worry about losing control of my bowels. | **□** | **□** | **□** | **□** | **□** |  |
| **32.** | I fear that I won’t be able to have a bowel movement. | **□** | **□** | **□** | **□** | **□** |  |
| **33.** | My abdominal/bowel problems are affecting my closest relationships. | **□** | **□** | **□** | **□** | **□** |  |
| **34.** | I feel that no-one understands my abdominal/bowel problems | **□** | **□** | **□** | **□** | **□** |  |

**SECTION 4.**

**This section asks about the ways in which abdominal and bowel symptoms affect your life and the things that are most important to you about treatment for these symptoms. If you no longer experience these symptoms please just write ‘No symptoms’ or ‘Not applicable’ for these questions.**

1. **In what ways do abdominal or bowel symptoms affect your everyday life?**

|  |  |  |  |  |
| --- | --- | --- | --- | --- |
|  |  |  |  |  |

1. **Are there things that you would like to do that you are not able to do because of abdominal or bowel symptoms?**

|  |  |  |  |  |
| --- | --- | --- | --- | --- |

1. **How do your abdominal and bowel symptoms make you feel emotionally?**

|  |  |  |  |  |
| --- | --- | --- | --- | --- |

1. **Which symptom affects you most?**

|  |  |  |  |  |
| --- | --- | --- | --- | --- |

1. **What aspects of your abdominal or bowel problems would you most like to see an improvement in? (This may be symptoms or your ability to do something or feel differently)**

|  |  |  |  |  |
| --- | --- | --- | --- | --- |

1. **What one thing would you most like to see improved?**

|  |  |  |  |  |
| --- | --- | --- | --- | --- |

**SECTION 5.**

**This section asks about the types of things you would be prepared to try to help manage your abdominal or bowel symptoms.**

**The treatments below are all things which may be useful for some people in treating abdominal and bowel problems. For each treatment please think about whether you would be likely to accept the treatment:**

**a) if your doctor offered it to you as part of your medical care**

**b) if your doctor suggested you have the treatment as part of a research study**

**c) if you had to arrange and pay for the treatment yourself**

|  | **Would you consider this treatment if your doctor suggested it as part of your medical care?**  **(Column 1)** | **Would you consider this treatment if your doctor suggested it as part of a research study?**  **(Column 2)** | **Would you consider this treatment if you had to arrange and pay for it yourself?** |
| --- | --- | --- | --- |
| A medication taken in tablet form | Yes **□** No **□** | Yes **□** No **□** | Yes **□** No **□** |
| *If you answered ‘No in column 1 or 2 above please tell us why you would not consider the treatment as part of your medical care or research study* | | | |
| A series of exercises similar to yoga | Yes **□** No **□** | Yes **□** No **□** | Yes **□** No **□** |
| *If you answered ‘No in column 1 or 2 above please tell us why you would not consider the treatment as part of your medical care or research study* | | | |
| A diet change (such as reducing fat or dairy products) | Yes **□** No **□** | Yes **□** No **□** | Yes **□** No **□** |
| *If you answered ‘No in column 1 or 2 above please tell us why you would not consider the treatment as part of your medical care or research study* | | | |

|  | **Would you consider this treatment if your doctor suggested it as part of your medical care?**  **(Column 1)** | **Would you consider this treatment if your doctor suggested it as part of a research study?**  **(Column 2)** | **Would you consider this treatment if you had to arrange and pay for it yourself?** |
| --- | --- | --- | --- |
| Acupuncture (a therapy which uses needles) | Yes **□** No **□** | Yes **□** No **□** | Yes **□** No **□** |
| *If you answered ‘No in column 1 or 2 above please tell us why you would not consider the treatment as part of your medical care or research study* | | | |
| Hypnotherapy (a therapy which involves going into a state of relaxation) | Yes **□** No **□** | Yes **□** No **□** | Yes **□** No **□** |
| *If you answered ‘No in column 1 or 2 above please tell us why you would not consider the treatment as part of your medical care or research study* | | | |
| A medication applied to the stomach in cream form | Yes **□** No **□** | Yes **□** No **□** | Yes **□** No **□** |
| *If you answered ‘No in column 1 or 2 above please tell us why you would not consider the treatment as part of your medical care or research study* | | | |
| Homeopathy (an alternative medicine prescribed by a homeopath) | Yes **□** No **□** | Yes **□** No **□** | Yes **□** No **□** |
| *If you answered ‘No in column 1 or 2 above please tell us why you would not consider the treatment as part of your medical care or research study* | | | |

|  | **Would you consider this treatment if your doctor suggested it as part of your medical care?**  **(Column 1)** | **Would you consider this treatment if your doctor suggested it as part of a research study?**  **(Column 2)** | **Would you consider this treatment if you had to arrange and pay for it yourself?** |
| --- | --- | --- | --- |
| A suppository medication which had to be inserted into the rectum | Yes **□** No **□** | Yes **□** No **□** | Yes **□** No **□** |
| *If you answered ‘No in column 1 or 2 above please tell us why you would not consider the treatment as part of your medical care or research study* | | | |
| A heat pad which stuck to the skin on your stomach (like a large plaster) and generated warmth to help sooth symptoms | Yes **□** No **□** | Yes **□** No **□** | Yes **□** No **□** |
| *If you answered ‘No in column 1 or 2 above please tell us why you would not consider the treatment as part of your medical care or research study* | | | |

**Would you be willing to discuss your symptoms and opinions in more detail?**

We would like to contact some people with different types of symptoms or opinions to discuss these in more detail. If you would be willing for a medical researcher to contact you to arrange an interview (at home or in your GP surgery) about your symptoms please could you tick the box below and provide your telephone number and best time to contact you.

**I would be willing to speak to a researcher about my symptoms and opinions about abdominal and bowel symptoms. Please contact me to arrange a convenient time:**

Yes **□** My telephone number is:_______________________________

The best time to contact me is:___________________________

**I would prefer not to be contacted to discuss my symptoms in more detail (tick the box below)**

No **□**

**Would you provide permission for a researcher from the Department of Primary Care at the University of Birmingham to review your medical notes (only notes held at your GP practice)?**

To help us to better understand why some people recover from episodes of irritable bowel syndrome and others continue to experience symptoms, we need to compare the medical records of patients who do and do not continue to experience symptoms. This will allow us to look at whether factors such as referral to a hospital doctor, use of certain medications (for IBS or for other illnesses) and other illnesses themselves have an impact on the course of IBS.

If you are willing to provide permission for a researcher to review your records you should tick all of the boxes below and sign and date this page.

**Consent form for medical note review**

IBS 2006 Study

| 1. I confirm that I have read and understood the information sheet dated 01/11/05 for the note review aspect of the above study. | **□** |
| --- | --- |
| 2. I understand that the decision to participate is voluntary and I am free to withdraw my consent at any time without giving any reason, without my medical care or legal rights being affected. | **□** |
| 3. I understand that sections of my medical notes will be looked at by responsible individuals from the Department of Primary Care at the University of Birmingham or from regulatory authorities where it is relevant to my taking part in research. I give permission for these individuals to have access to my records. | **□** |
| 4. I agree to take part in this study. | **□** |

| **Print Name:** | **_____________** | **Date:** | **___________** | **Signature:** | **________________** |
| --- | --- | --- | --- | --- | --- |

| Thank you very much for completing this questionnaire.  The information which you have provided will help us to understand more about the effects of abdominal and bowel symptoms and ensure future work in this area takes patients views into account.  Thank you for your co-operation.  Please return this questionnaire in the **FREEPOST** envelope provided.  No stamp is required. |
| --- |
